# From screens to minds: the mediating role of psychological well-being between digital reading and AI anxiety

**DOI:** 10.3389/fpsyg.2025.1727759

**Published:** 2025-12-10

**Authors:** Uğur Özbilen, Emrullah Banaz, Tuğrul Gökmen Şahin

**Affiliations:** 1 Independent Researcher, Antalya, Türkiye; 2 Department of Educational Sciences, Bayburt University, Bayburt, Türkiye; 3 Department of Social Sciences and Turkish Language Education, Inonu University, Malatya, Türkiye

**Keywords:** digital reading disposition, psychological well-being, artificial intelligence anxiety, structural equation modeling, teacher education

## Abstract

**Introduction:**

This study investigates the mediating role of psychological well-being in the relationship between digital reading disposition and artificial intelligence (AI) anxiety among Turkish teachers. Addressing the growing concern of technology-related anxiety in education, the research explores how digital literacy and psychological resilience interact within a single structural model.

**Methods:**

A correlational research design was employed with data collected from 324 teachers. Participants completed the Digital Reading Disposition Scale, the Psychological Well-Being Scale, and the Artificial Intelligence Anxiety Scale. Bootstrapped structural equation modeling (SEM) was used to test the measurement model and the hypothesized mediation effect. Prior to SEM analysis, confirmatory factor analysis (CFA) was conducted to establish construct validity of the scales.

**Results:**

Findings revealed that digital reading disposition was positively associated with psychological well-being and negatively linked with AI anxiety. Psychological well-being was also negatively related to AI anxiety. Moreover, psychological well-being partially mediated the relationship between digital reading disposition and AI anxiety.

**Discussion:**

The results suggest that teachers with stronger digital reading dispositions experience higher psychological well-being, which in turn buffers against AI-related anxiety. These findings contribute novel theoretical insights to educational literature by integrating digital reading, psychological well-being, and AI anxiety into a single model. Practically, the study underscores the importance of fostering digital literacy and psychological resilience to mitigate technology-related anxiety in educational settings.

## 1 Introduction

The ways individuals access information and engage in learning are undergoing a profound transformation in today’s digitalizing world. Printed materials are increasingly being replaced by digital content (Nyambane, 2021; Furenes et al., 2021; Hongcharu, 2024), which reshapes the very nature of reading and reading-related practices. Reading is no longer an interaction confined to printed texts; rather, it has evolved into a multilayered process that integrates hyperlinks, audiovisual materials, and interactive platforms. This shift has altered how individuals focus, process information, and form learning habits while simultaneously highlighting the importance of digital reading disposition. Digital reading disposition has emerged as a key construct shaping one’s engagement with online information, motivation, preferences, and reading strategies (Vargas, 2024; Kucirkova, 2022). Accordingly, understanding the cognitive and emotional dynamics of digitalization has become a central pursuit in contemporary educational research.

Artificial intelligence (AI) technologies now assume an increasingly central role in everyday life. However, such a tremendous evolution in technology carries the potential to provoke anxiety by disrupting one’s established sense of security and stability (Müller and Bostrom, 2014). Defined as the unease stemming from uncertainty and perceived loss of control over AI (Ryan, 2023), AI anxiety has become salient in the context of rapidly evolving generative AI applications. These applications often trigger future-related concerns among the young, including fears of shifting professional roles or inadequacy in adapting to new technologies. Larson (2021) suggests that this phenomenon stems not only from fears of unemployment but also from the “black box” nature of AI systems—that is, the opacity of their decision-making processes. Similarly, Fast and Horvitz (2017) emphasize that uncertainty regarding AI’s long-term consequences is one key driver of AI anxiety. Ryan (2023) further argues that the extent to which individuals experience such anxiety varies depending on factors such as technological literacy and psychological resilience. Recent studies, in turn, have demonstrated that psychological well-being may serve as a buffer against technology-related anxiety and enable individuals to cope more effectively with the stress associated with AI and digital transformation (Cengiz, 2025; Li, 2025).

Psychological well-being refers to a holistic state of wellness encompassing self-perception, sense of meaning in life, environmental mastery, and autonomy. It assumes a critical role in one’s capacity to cope with stress (Ryff, 1989). This construct may function as a mediating mechanism in the relation between digital reading disposition and AI anxiety. Testing the mediating role of psychological well-being can shed light on the indirect effects of digital literacy tendencies on one’s technological anxiety. Indeed, digital reading disposition can enhance psychological well-being by helping individuals manage their cognitive resources more efficiently, maintain their learning motivation, and reinforce their sense of self-efficacy in accessing information (Sun et al., 2022). Individuals with higher levels of psychological well-being, in turn, were previously shown to display greater resilience toward technological innovation and to respond more flexibly to uncertainty and anxiety (Yazıcı Çelebi et al., 2025). In this sense, the buffering role of psychological well-being may help mitigate the adverse effects of AI anxiety through the cognitive and emotional gains associated with digital reading disposition. Although existing literature hosts studies that have already examined each of these variables independently, research that integrates them into a single structural model remains scarce. Therefore, the proposed mediation model is believed to offer not only novel theoretical insights into educational literature but also practical implications for educational policy, digital pedagogy, and psychological counseling practices (Zakir, 2025; Vargas, 2024; Li, 2025). Overall, this study provides an original contribution by elucidating the opportunities and challenges of the digital age through the lens of individual psychological processes.

### 1.1 Digitalization and Reading dispositions

Digitalization has not only shifted reading practices from paper to screen but also fundamentally transformed how individuals engage with texts, learn, and access information (Liao et al., 2024; Salmerón et al., 2023). The portability, low cost, convenient access, and multimodal formats offered by digital environments diversify reading experiences and, particularly in educational contexts, facilitate access to learning materials (Pew Research Center, 2022). However, reading preferences cannot be reduced solely to technological factors; they are also tailored by the type of text, learning purpose, individual habits, and pedagogical context (Baron, 2021).

Digital reading involves not only a shift in the physical aspect of text but also a reorganization of cognitive and behavioral processes. Hypertext, multimedia elements, and instant access offer readers unprecedented richness and convenience; yet, they can also lead to a state of continuous partial attention, which challenges sustained deep focus (Wolf and Barzillai, 2009). In particular, superficial reading strategies (e.g., skimming and scanning) have emerged as a natural byproduct of digital environments and potentially hinder interaction with texts that demand critical thinking and deeper comprehension.

The ways one interacts with digital texts play a decisive role in shaping their digital reading disposition. For example, the structure of hypertext allows readers to establish connections between texts, explore information in a multilayered manner, and diversify their individual learning pathways (DeStefano and LeFevre, 2007). Moreover, the use of multimodal content enriches texts with visual, auditory, and interactive elements, adding both cognitive and emotional depth to the learning process (Mangen et al., 2019). In addition, digital reading platforms (e.g., Wattpad, Radish, and Inkitt) transform reading from a solely individual activity into a socially interactive experience. Readers can share comments, participate in collaborative discussions, and engage in community-based meaning-making through these platforms (Rowberry, 2016). These processes, therefore, allow reading to be redefined as not only an engagement with text but also an interactive, multisensory, and community-oriented learning practice.

In sum, the impact of digitalization on reading processes represents not merely a technological shift but a complex phenomenon redefined across cognitive, pedagogical, and social dimensions (Clinton, 2019; Singer and Alexander, 2017). The flow of multiple stimuli in digital settings, hypertext structures, and constant transitions can fragment attention and undermine sustained focus; in some contexts, this reinforces superficial reading strategies while hindering deep comprehension (Delgado et al., 2018; Firth et al., 2019). Combined with the information overload and cognitive demands commonly encountered in digital settings, these patterns can trigger anxiety (Jiang et al., 2022).

The cognitive overload and anxiety associated with digital reading provide an important foundation for shaping individuals’ attitudes and concerns toward new and increasingly complex technological developments (Singer and Alexander, 2017; Venkatesh, 2000). In this framework, it becomes evident that the anxiety stemming from digital reading experiences has evolved into what is now recognized as one of the most salient forms of technology-related concern: AI anxiety.

### 1.2 AI anxiety

Despite the widespread adoption of AI across education, healthcare, law, and social services, the rise of AI technologies has sparked apprehension among various segments of society, primarily due to the potential impacts of automation on employment, the societal implications of biased algorithms, and data privacy issues (Madaoui, 2024; Nie, 2023). For instance, the integration of AI in education can alter teaching processes and lead instructors to experience feelings of job insecurity (Popenici and Kerr, 2017). Similarly, the adoption of AI in healthcare challenges the role of human practitioners and raises concerns about its effects on patient outcomes (Murphy et al., 2021; Amedior, 2023). The increasingly automated nature of AI applications necessitates the restructuring of the workforce and amplifies factors that affect employees’ psychological well-being (Rubeis, 2022). Moreover, AI-driven systems in social services, justice, and healthcare provoke critical debates about potential discrimination and bias in their application (Klímová et al., 2023).

AI-related concerns are multidimensional, encompassing ethical, social, and economic facets with a range of implications. Concerns such as potential workforce displacement, data privacy, and the influence of AI on decision-making processes underscore the need for comprehensive research and policy development to understand the societal impact of these technologies. Defining a clear framework for AI’s societal role is pivotal for ensuring sustainable and well-coordinated development across sectors, including education and healthcare (Nie, 2023). In this sense, AI anxiety is associated with one’s experiences of uncertainty, perceived loss of control, and threat during technological transformations. However, the impact of this anxiety varies depending on one’s psychological resources and coping abilities. In particular, psychological well-being represents a fundamental construct that strengthens stress resilience and self-efficacy. Therefore, understanding AI anxiety requires attention not only to cognitive processes but also to emotional mechanisms.

### 1.3 Psychological well-being

Psychological well-being is an interdisciplinary concept applicable to individuals across all demographics and has recently emerged as a prominent focus of scholars. The concept encompasses one’s perception of life as meaningful, self-realization, positive relationships with others, and effective functioning in daily life. Ryff and Keyes’s (1995) psychological well-being model and Seligman’s (2018) PERMA model are particularly prominent when it comes to addressing psychological well-being. These frameworks provide key insights into the ways psychological well-being shapes individual functioning. At its core, the concept of psychological well-being relies on fundamental functional dimensions, including resilience, autonomy, life satisfaction, and environmental mastery (Ryff and Keyes, 1995; Seligman, 2018).

One’s self-efficacy, personal growth, authentic identity, social relationships, and sense of purpose in life are highly emphasized in Ryff’s psychological well-being model (Kim et al., 2011). It is often suggested that these dimensions may be further enhanced when reinforced by individual factors such as spiritual orientation. Previous research also indicated that individuals with higher levels of psychological well-being tend to cope more effectively with psychological challenges (Coppola et al., 2021). Seligman’s PERMA model offers a more systematic framework for conceptualizing this construct. This model conceptualizes psychological well-being as the interaction of five core components—positive emotion, engagement, relationships, meaning, and accomplishment (Nasser and Fakhroo, 2021). It also posits that spiritual orientation can help one find meaning in life and enhance their psychological well-being (Ríos-Risquez et al., 2018). Spiritual experiences allow one to cultivate positive emotions toward diverse aspects of life, establish deeper social connections, and maintain resilience in the face of adversity (Tang et al., 2016). It was previously demonstrated that spiritual orientation positively influences one’s overall happiness and life satisfaction (Jafari and Hesampour, 2017). In today’s context, psychological well-being both affects and is affected by a wide range of variables, among which technology plays a prominent role.

The relation between attitudes toward technology and psychological well-being has become an increasingly prominent area of inquiry, particularly with the rapid growth of digitalization. For instance, the integrated use of information and communication technologies (ICT) in educational settings was found to enhance students’ psychological well-being by addressing their emotional needs (Vistorte et al., 2024). In this regard, one’s emotional responses and feelings toward technology significantly influence both their acceptance of technological innovations and accompanying benefits (Lin et al., 2024). Moreover, there is robust evidence indicating that positive emotions contribute to the development of more favorable attitudes toward technology (Wu et al., 2021), which, in turn, affects one’s overall psychological well-being (Timmons et al., 2022).

**Figure 1:**
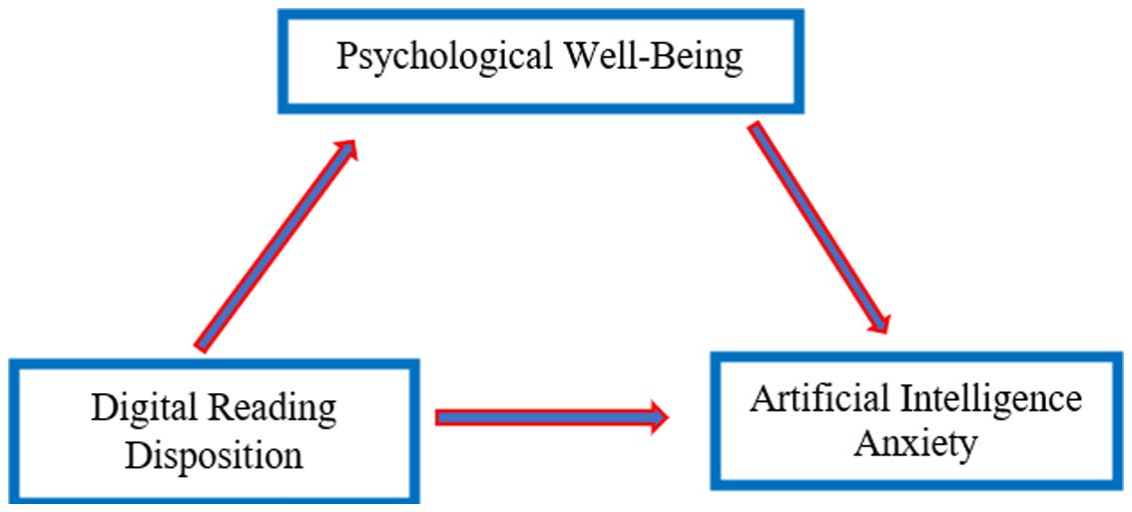
Proposed SEM model.

Digitalized literacy is reshaping one’s cognitive and emotional orientations. It not only encourages more conscious and critical engagement with technology but also has the potential to strengthen psychological resilience. In this sense, growing evidence suggests that digital self-efficacy and digital literacy positively contribute to psychological well-being (Buckingham et al., 2023; Kong and Liu, 2023; Xin et al., 2025). Conversely, AI anxiety has emerged as a new source of psychological stress (Kim et al., 2025; Kaya et al., 2022). Psychological well-being, however, may serve as a buffering mechanism against such anxiety, thereby playing a critical mediating role in the relation between digital reading disposition and AI anxiety. Examining this mediating role not only addresses a notable gap in the current literature but also provides deeper insights into how technological transformations shape one’s psychological experiences. Based on this theoretical framework, the proposed conceptual model is presented in Figure 1 and tested through the hypotheses outlined below.

*H1*: Digital reading disposition is positively associated with psychological well-being.

*H2*: Psychological well-being is negatively associated with AI anxiety.

*H3*: Digital reading disposition is negatively associated with AI anxiety.

*H4*: Psychological well-being mediates the relation between digital reading disposition and AI anxiety.

## 2 Method

In this study, we employed a correlational design. It allows for quantitatively describing overall trends, attitudes, and perceptions based on the data collected from a sample representing a specific population (Creswell, 2016). The primary goal of correlational research is to determine whether variables vary together and, if so, to identify the direction and nature of relations (Karasar, 2011). The rationale for adopting this design in this study lies in its capacity to provide a deeper understanding of both the direct and indirect associations among variables (Gliner et al., 2017).

### 2.1 Sample

The sample consists of teachers employed in public schools. The majority of them were females (63.3%), while male teachers accounted for about one-third of the sample (36.7%). The group was composed of both novice and experienced teachers. Regarding educational background, most of the participating teachers held a bachelor’s degree (65.4%), while a considerable proportion had completed graduate-level education (34.6%). These findings are presented in detail in Table 1.

**Table 1:** Participants’ demographics.

Characteristic		*n*	%
Gender	Male	119	36.7
	Female	205	63.3
Professional seniority (years)	1–5	81	25.0
	6–10	56	17.3
	11–15	83	25.6
	>15	104	32.1
Education	Undergraduate	212	65.4
	Postgraduate	112	34.6

### 2.2 Data collection tools

#### 2.2.1 Psychological Well-Being Scale (PSWBS)

In this study, the 8-item Psychological Well-Being Scale, originally developed by Diener et al. (1985) and adapted into Turkish by Telef (2013), was employed to assess individuals’ levels of psychological well-being. The scale uses a 7-point Likert-type rating system ranging from “Strongly disagree” (1) to “Strongly agree” (7). Higher total scores on the scale indicate that individuals possess various psychological resources and strengths. While the adaptation study conducted by Telef (2013) reported an internal consistency coefficient of 0.80, the internal consistency coefficient in the present research was calculated as 0.87.

#### 2.2.2 Digital Reading Disposition Scale (DRDS)

Bulut and Karasakaloğlu (2018) designed the DRDS to assess pre-service teachers’ digital reading disposition. It is a single-factor instrument consisting of 12 items designed to evaluate participants’ interest, motivation, and tendencies in reading digital texts. Responses are rated on a 5-point Likert-type scale (1 = Does not fit me at all, 5 = Fits me at all). Higher scores indicate a more positive digital reading disposition. In the original study, the internal consistency was reported as 0.92. In this study, we calculated Cronbach’s alpha coefficient for the total DRDS score to be 0.85. Four items were removed from the original scale to enhance conceptual coherence and strengthen the instrument’s psychometric properties. These items showed low factor loadings and weak item–total correlations in the preliminary analyses, indicating that they did not sufficiently contribute to the unidimensional structure of the scale.

#### 2.2.3 Artificial Intelligence Anxiety Scale (AIAS)

The AIAS was originally developed by Wang and Wang (2022) and adapted into Turkish by Akkaya et al. (2021). The scale consists of 16 items within four subscales: learning, job replacement, sociotechnical blindness, and AI configuration. Items are rated on a 7-point Likert-type scale (1 = Strongly Disagree, 7 = Strongly Agree). In the adaptation study, the authors reported Cronbach’s alpha coefficients to be 0.937 for the total AIAS score and between 0.875 and 0.950 for the subscales. In the present study, we computed Cronbach’s alpha for the total AIAS score to be 0.95.

### 2.3 Data analysis

The present study explores the mediating role of psychological well-being in the association between digital reading disposition and AI anxiety among public school teachers in Türkiye. The data are summarized as number (*n*), percentage (%), mean (*M*), and standard deviation (*SD*). Before the analyses, we checked outliers, missing data, and univariate normality (skewness-kurtosis values; Çokluk et al., 2021). Consequently, the data from 32 participants were excluded due to their status as outliers, and the analyses were performed on the data from 324 participants. The relations between the research variables were first explored using Pearson’s correlation analysis. Then, the variables were recruited for structural equation modeling (SEM) to test the measurement model and research hypotheses. It is worth noting that all measurement tools were first subjected to confirmatory factor analysis (CFA) to test their construct validity and ensure a robust test of the measurement model. We performed all analyses on SPSS 27.0 and SPSS AMOS 24.0 and accepted a *p*-value of < 0.05 as statistically significant.

## 3 Findings

Initially, we tested the construct validity of the instruments used in this study to ensure a robust evaluation of the measurement model. The initial CFA for the DRDS showed that the regression weights of four items fell well below the critical threshold of 0.40. When these items were removed from the theoretical model one by one, the remaining eight items demonstrated acceptable regression weights, ranging from 0.427 to 0.689. In the final analysis, the model demonstrated an acceptable fit to the data. In the CFA performed for the PSWBS, the regression weights of eight items ranged between 0.446 and 0.688. The model also demonstrated an acceptable fit with the data. Finally, the CFA performed for the AIAS showed that the regression weight of one item from the AI Configuration subscale fell below the critical threshold. After this item was removed from the model, the remaining items showed regression weights ranging from 0.639 to 0.968. The results also showed an acceptable model-data fit for the AIAS. The suggested thresholds for fit indices and our results are presented in Table 2. Overall, the subsequent analyses were performed based on participant scores from the respective scales whose construct validity was confirmed through the CFA results.

**Table 2:** Model-data fit indices of the measurement tools.

Index	Good fit	Acceptable fit	DRDS	PSWBS	AIAS
*χ*^2^/df	*χ*^2^/df ≤ 3	*χ*^2^/df ≤ 5	2.562	2.795	3.241
RMSEA	RMSEA ≤ 0.05	RMSEA ≤ 0.08	0.070	0.075	0.083
CFI	CFI ≥ 0.95	CFI ≥ 0.90	,904	0.942	0.966
NFI	NFI ≥ 0.95	NFI ≥ 0.90	0.855	0.914	0.951
GFI	GFI ≥ 0.95	GFI ≥ 0.90	0.963	0.960	0.910
TLI	TLI ≥ 0.95	TLI ≥ 0.90	0.858	0.910	0.954
AGFI	AGFI ≥ 0.90	AGFI ≥ 0.85	0.929	0.921	0.861

Çokluk et al. (2021); Hu and Bentler (1999); Kline (2005); Schumacker and Lomax (2004); Sümer (2000); Tabachnick and Fidell (2013). *χ*^2^/df, Chi-square/degrees of freedom, RMSEA, root mean square error of approximation, CFI, comparative fit index, NFI: normed fit index, GFI, goodness-of-fit index, TLI: Tucker–Lewis index, AGFI: adjusted GFI.

**Figure 2:**
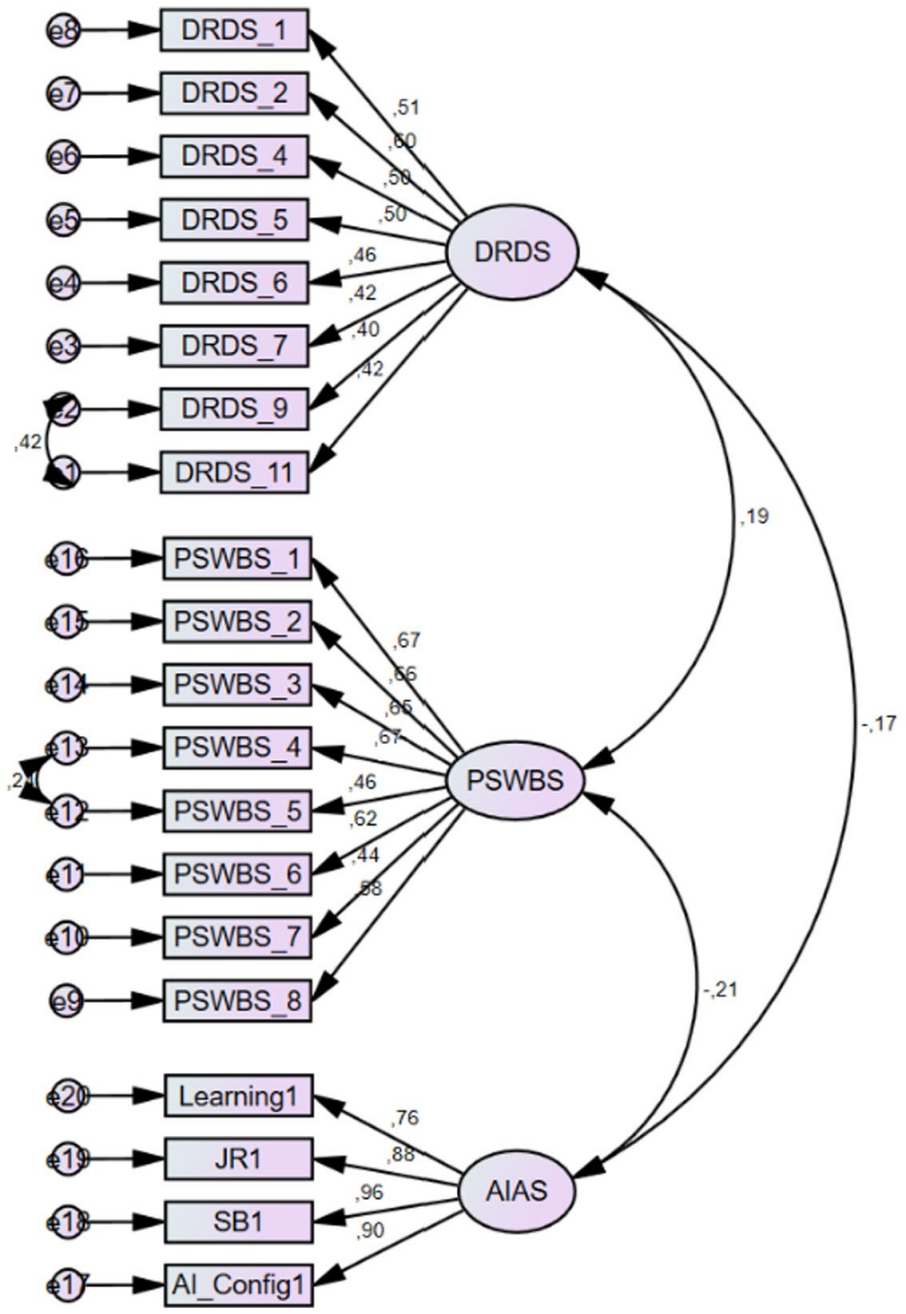
Measurement model. DRDS: Digital Reading Disposition Scale, PSWBS: Psychological Well-Being Scale, AIAS: Artificial Intelligence Anxiety Scale, JR: job replacement, SB: sociotechnical blindness, AI_Config: AI configuration.

Prior to exploring the potential influence of common method bias, we ran an exploratory factor analysis (EFA) with principal axis factoring on all items covered by the instruments. The results showed 7 factors with eigenvalues exceeding 1. The initial eigenvalue for the first factor was 9.601 that accounts for 30.97% of the variance. As this value fell below the critical threshold of 40%, we may assert that our findings would be free of common method bias (Podsakoff et al., 2003). In the measurement model testing, we ran another CFA with the respective variables. The results showed that the regression weights in the model ranged between 0.40 and 0.96 (*t* > 2.58, *p* < 0.01). In addition, we found acceptable model-data fit indices for our measurement model (*χ^2^*/df = 1.792, RMSEA = 0.050, CFI = 0.936, NFI = 0.868, GFI = 0.915, TLI = 0.927, AGFI = 0.892). The tested measurement model is depicted in Figure 2.

We present descriptives of participant scores, univariate normality findings, and the results of the correlation analysis (Table 3). As the data exhibited a normal distribution (skewness and kurtosis values ≤ ±1; Çokluk et al., 2021), we calculated Pearson’s correlation coefficients and found digital reading disposition to exhibit weak correlations with psychological well-being (*r* = 0.129, *p* = 0.021) and AI anxiety (*r* = −0.158, *p* = 0.004). There was also a weak negative association between psychological well-being and AI anxiety (*r* = −0.208, *p* < 0.01). Moreover, we examined multicollinearity among the latent variables using VIF values. A VIF below 5 combined with a tolerance above 0.20 indicates the absence of multicollinearity (Hair et al., 2014). In line with these criteria, the calculated statistics for the independent and dependent variables were 1.045 and 0.957, respectively, confirming that multicollinearity was not a concern in this study.

**Table 3:** Descriptive statistics and findings of the correlation analysis.

Variables	*n*	Min.	Max.	*M*	*SD*	Skewness	Kurtosis	Correlations		
								1	2	3
1. Digital reading disposition	324	16.00	38.00	27.89	4.57	−0.152	−0.416	1	0.129*	−0.158*
2. Psychological well-being	324	34.00	56.00	46.44	4.76	−0.333	−0.013		1	−0.208*
3. AI anxiety	324	15.00	75.00	46.20	12.73	0.080	−0.380			1
Learning	324	5.00	25.00	13.43	4.59	0.319	−0.394			
Job replacement	324	4.00	20.00	13.54	3.68	−0.124	−0.414			
Sociotechnical blindness	324	4.00	20.00	13.05	3.66	−0.035	−0.398			
AI configuration	324	2.00	10.00	6.16	2.14	0.073	−0.710			

*Correlation is significant at the 0.05 level (2-tailed).

**Figure 3:**
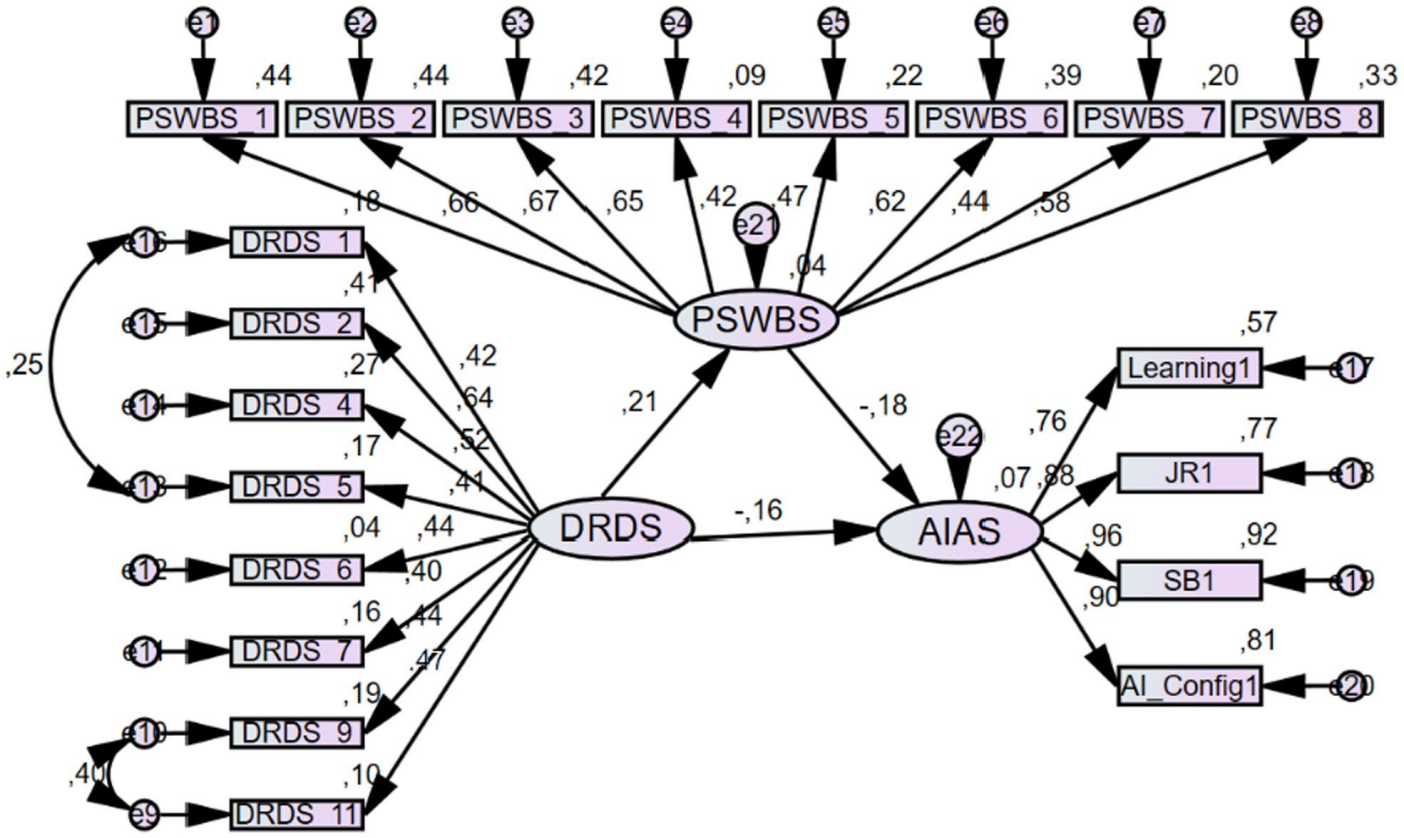
Conceptual framework of the mediating role of psychological well-being in the relation between digital reading disposition and AI anxiety.

The follow-up mediation analysis revealed the model to yield the following values for baseline goodness-of-fit indices: *χ^2^*/df = 1.807, *p* < 0.001, RMSEA = 0.050, NFI = 0.867, Relative Fit Index (RFI) = 0.846, Incremental Fit Index (IFI) = 0.936, TLI = 0.925, and CFI = 0.935. We found a significant negative relation between reading digital reading disposition on AI anxiety (*β* = −0.155, 95% Confidence Interval (CI): −0.440 to −0.043, *p* = 0.034). It was also found to have a significant positive association with psychological well-being (*β* = 0.210, 95% CI: 0.122–0.607, *p* = 0.010). Participants’ psychological well-being scores were negatively linked with their AIAS scores (*β* = −0.180, 95% CI: −0.278 to −0.059, *p* = 0.006). Therefore, the first three hypotheses of this study were confirmed. Moreover, the analysis revealed a significant indirect effect of psychological well-being on the relation between digital reading disposition and AI anxiety (*β* = −0.038, 95% CI: −0.119 to −0.017, *p* = 0.011), confirming our final hypothesis. Both the direct and indirect paths were significant, indicating partial complementary mediation (Zhao et al., 2010). Consistent with Baron and Kenny’s (1986) approach, the effect of digital reading disposition on AI anxiety decreased from −0.193 to −0.155 after including the mediator, while remaining statistically significant, further confirming partial mediation (Table 4). Figure 3 presents the conceptual framework of the mediating role of psychological well-being in the relation between digital reading disposition and AI anxiety.

**Table 4:** Mediating role of psychological well-being in the relation between digital reading disposition and AI anxiety.

Type	Effect	Estimate	SE	95% CI (a)		*β*	*p*
				Lower	Upper		
Indirect	Digital reading disposition ⇒ Psychological well-being ⇒ AI anxiety	−0.057	0.031	−0.119	−0.017	−0.038	0.011*
Component	Digital reading disposition ⇒ Psychological well-being	0.342	0.133	0.122	0.607	0.210	0.010*
	Psychological well-being ⇒ AI anxiety	−0.166	0.061	−0.278	−0.059	−0.180	0.006*
Direct	Digital reading disposition ⇒ AI anxiety	−0.233	0.120	−0.440	−0.043	−0.155	0.034*
Total	Digital reading disposition ⇒ AI anxiety	−0.290	0.123	−0.519	−0.096	−0.310	0.018*

Confidence intervals computed with the method: Bootstrap percentiles.

Betas indicate standardized effect sizes. *p < 0.05.

## 4 Discussion and conclusions

This study aimed to explore the relations between teachers’ digital reading disposition, psychological well-being, and AI anxiety, while also examining the mediating role of psychological well-being within these connections. The findings revealed that, beyond the direct associations observed among the variables, psychological well-being served as a key mediating construct. In particular, the cognitive and affective resources that teachers cultivate through their digital reading experiences appear to exert an influence on their AI anxiety. Collectively, these outcomes yield original insights of both theoretical and practical relevance. Drawing on these results, we addressed each of the hypotheses tested in relation to the broader scholarly discourse.

The findings confirmed a significant positive effect of digital reading disposition on psychological well-being. This result suggests that digital literacy practices are likely to contribute not only to cognitive processes but also to several aspects of psychological well-being, such as self-efficacy (Siddiq et al., 2017), environmental mastery (i.e., the perceived sense of control over online environments), and life satisfaction (Ryff, 1989; Heeter et al., 2011; Van Deursen and Van Dijk, 2019). Underlying this relation are the diverse features of digital reading. For instance, the hypertextual transitions characteristic of digital environments provide opportunities for exploration and control and foster learning motivation (Conklin, 1987). Likewise, multimodal elements in digital texts facilitate more efficient cognitive processing and promote deeper engagement with information (Mayer, 2014). Moreover, when digital reading disposition becomes habitual, it can cultivate more reflective and conscious attitudes toward technology and strengthen positive affect and a sense of meaning.

Our model uncovered a significant negative link between psychological well-being and AI anxiety. We can, therefore, infer that those with higher levels of psychological well-being become more resilient when facing technological uncertainty, perceived loss of control, or professional transformations and can manage stressors more efficiently. The literature consistently emphasizes that psychological well-being serves as a buffering mechanism against technology-related anxiety (Asad et al., 2023; Wang and Yao, 2025; Consiglio et al., 2023). Highly resilient individuals appear better equipped to cope with the unpredictable risks arising from the rapid development of AI. The relevant evidence also indicates that cognitive flexibility and problem-solving skills under conditions of uncertainty help reduce anxiety (Yazıcı Çelebi et al., 2025). Taken together, our findings highlight that psychological well-being not only fosters happiness and life satisfaction but also functions as a protective mechanism against the psychological pressures exposed by emerging technologies.

There was a significant negative relation between digital reading disposition and AI anxiety. This implies that one with higher digital literacy tends to approach AI technologies more thoughtfully and critically, which may reduce their susceptibility to uncertainty and feelings of lost control. Previous studies similarly highlighted that digital competence and self-efficacy can mitigate technology-related anxieties (Şimşek, 2011; Paul, 2017; Achim and Al Kassim, 2015). Moreover, technology acceptance models emphasize that perceived ease of use and self-regulation play a critical role in shaping technology-related anxiety (Venkatesh, 2000). In this context, our findings suggest that a strong digital reading disposition may enhance both cognitive and emotional resources; therefore, it may serve a protective function against AI anxiety. These results also underscore the significance of digital literacy, particularly in teaching, as a key factor in alleviating technology-induced anxiety.

This study revealed a partial mediating role of psychological well-being in the relation between digital reading disposition and AI anxiety. Hence, digital literacy practices may not only help lower AI-related anxiety but also indirectly support psychological well-being, which buffers individuals against technology-related stressors. In other words, digitally competent individuals appear better equipped to cope with uncertainties and perceived loss of control associated with AI technologies.

The relevant literature also highlights that psychological well-being can mediate the positive effects of digitalization on mental health (Carlbring et al., 2018; Firth et al., 2017) and enable individuals to develop healthier coping mechanisms against technological stress through their psychological resources (Tarafdar et al., 2019; Salanova et al., 2013). Our finding confirms that AI anxiety is not merely shaped by cognitive or demographic factors but is also closely tied to one’s psychological experiences and inner strengths. Therefore, the partial mediation model proposed in this study offers meaningful practical implications for educational policy, teacher training programs, and psychological counseling practices. In particular, designing interventions that aim to enhance teachers’ digital reading skills while simultaneously fostering their psychological well-being may create a more holistic and sustainable impact in reducing their AI-related anxiety.

Overall, our study provides a multidimensional understanding of the relations among teachers’ digital reading disposition, psychological well-being, and AI anxiety. The findings demonstrate that digital reading disposition can not only enhance cognitive processes but also promote psychological well-being and reduce AI anxiety both directly and indirectly. In particular, the partial mediating role of psychological well-being highlights that individual with stronger psychological resources tend to exhibit greater resilience and adaptability when facing technological uncertainties. Accordingly, fostering teachers’ digital competencies alongside their psychological well-being may serve as an effective and sustainable strategy for strengthening their professional adaptability in the context of ongoing technological transformation.

## 5 Limitations and future directions

This study is not free of a few limitations. Its cross-sectional nature precludes drawing causal inferences. In addition, we collected the data through self-reports from Turkish teachers, which may limit the cross-cultural generalizability of the findings. Considering potential differences in participants’ technology use experiences, years of professional experience, and subject areas, our research should be replicated with more diverse and representative samples.

Future studies could address these limitations by employing longitudinal and experimental designs to examine the long-term effects of teachers’ digital reading practices on psychological well-being and AI anxiety. Moreover, intervention-based endeavors may test how initiatives to enhance digital literacy skills within teacher training programs influence teachers’ psychological well-being and adaptation to technology. Qualitative studies may also provide deeper insights into how teachers use digital reading tools in the classroom, the pedagogical strategies they adopt, and how they cope with technology-related anxiety. Cross-cultural comparisons involving teacher samples from different countries could offer a broader understanding of how education systems respond to digitalization and AI integration. Finally, future research that incorporates pedagogical context may contribute to the development of more effective, sustainable, and psychologically supportive approaches in both teacher training and school-based policy design.

## Data Availability

The raw data supporting the conclusions of this article will be made available by the authors, without undue reservation.
