# Correction: From screens to minds: the mediating role of psychological well-being between digital reading and AI anxiety

**DOI:** 10.3389/fpsyg.2025.1771451

**Published:** 2026-01-12

**Authors:** Uǧur Özbilen, Emrullah Banaz, Tuǧrul Gökmen Şahin

**Affiliations:** 1 Independent Researcher, Antalya, Türkiye; 2 Department of Educational Sciences, Bayburt University, Bayburt, Türkiye; 3 Department of Social Sciences and Turkish Language Education, Inonu University, Malatya, Türkiye

**Keywords:** digital reading disposition, psychological well-being, artificial intelligence anxiety, structural equation modeling, teacher education

Affiliation 3 “Department of Social Sciences and Turkish Language Education, Inonu University, Malatya, Türkiye” was erroneously given as “Department of Social Sciences and Turkish Language Education, Ataturk University, Erzurum, Türkiye”.

The original version of this article has been updated.

